# Tobacco Product Use Among Middle and High School Students — United States, 2011 and 2012

**Published:** 2013-11-15

**Authors:** René A. Arrazola, Shanta R. Dube, Brian A. King

**Affiliations:** Office on Smoking and Health, National Center for Chronic Disease Prevention and Health Promotion, CDC

Nearly 90% of adult smokers in the United States began smoking by age 18 years (1). To assess current tobacco product use among youths, CDC analyzed data from the 2012 National Youth Tobacco Survey (NYTS). This report describes the results of that analysis, which found that, in 2012, the prevalence of current tobacco product use among middle and high school students was 6.7% and 23.3%, respectively. After cigarettes, cigars were the second most commonly used tobacco product, with prevalence of use at 2.8% and 12.6%, respectively. From 2011 to 2012, electronic cigarette use increased significantly among middle school (0.6% to 1.1%) and high school (1.5% to 2.8%) students, and hookah use increased among high school students (4.1% to 5.4%). During the same period, significant decreases occurred in bidi* and kretek† use among middle and high school students, and in dissolvable tobacco use among high school students. A substantial proportion of youth tobacco use occurs with products other than cigarettes, so monitoring and prevention of youth tobacco use needs to incorporate other products, including new and emerging products. Implementing evidence-based interventions can prevent and reduce tobacco use among youths as part of comprehensive tobacco control programs. In addition, implementation of the 2009 Family Smoking Prevention and Tobacco Control Act, which granted the Food and Drug Administration (FDA) the authority to regulate the manufacture, distribution, and marketing of tobacco products (1–3), also is critical to addressing this health risk behavior.

NYTS is a school-based, self-administered, pencil-and-paper questionnaire administered to U.S. middle school (grades 6–8) and high school (grades 9–12) students to collect information on key tobacco control outcome indicators used to monitor the impact of comprehensive tobacco control policies and programs (4) and FDA’s newly granted regulatory authority. NYTS was conducted in 2000, 2002, 2004, 2006, 2009, 2011, and 2012. The 2012 NYTS used a three-stage cluster sampling procedure to generate a cross-sectional, nationally representative sample of students in grades 6–12. This report includes 2011 and 2012 NYTS data to provide an updated definition of current tobacco use, which now also includes hookahs, snus, dissolvable tobacco, and electronic cigarettes, to take into account nonconventional products that are new to the market or are increasing in popularity; data for these four products were first collected in 2011. The previous definition for current tobacco use did not include all of these products, thus yielding slightly lower estimates of current tobacco use. For example, in 2011, the previous definition for overall current tobacco use resulted in estimates of 7.1% for middle school and 23.2% for high school students (5), whereas the new definition resulted in 2011 estimates of 7.5% for middle school and 24.3% for high school students (Table).

Of the 284 schools selected for the 2012 NYTS, 228 (80.3%) participated, resulting in a sample of 24,658 (91.7%) among 26,873 eligible students; the overall response rate was 73.6%. The 2011 NYTS had a comparable overall response rate of 72.7% (5). Respondents were asked about their current use of cigarettes, cigars§ (defined as cigars, cigarillos, or little cigars), smokeless tobacco, pipes, bidis, kreteks, hookahs, snus, dissolvable tobacco, and electronic cigarettes. For each product, current use was defined as using on ≥1 day of the past 30 days.

Data were adjusted for nonresponse and weighted to provide national prevalence estimates with 95% confidence intervals for current tobacco use overall and by product, school level, sex, and race/ethnicity. Point estimate differences between 2011 and 2012 were assessed using a two-tailed t-test for significance (p<0.05).

In 2012, 6.7% of middle students reported current use of any tobacco product (Table). The most commonly used forms of tobacco were cigarettes (3.5%), cigars (2.8%), pipes (1.8%), smokeless tobacco (1.7%), hookahs (1.3%), electronic cigarettes (1.1%), snus (0.8%), bidis (0.6%), kreteks (0.5%), and dissolvable tobacco (0.5%). Among high school students, 23.3% reported current use of any tobacco product. The most commonly used forms of tobacco were cigarettes (14.0%), cigars (12.6%), smokeless tobacco (6.4%), hookahs (5.4%), pipes (4.5%), electronic cigarettes (2.8%), snus (2.5%), kreteks (1.0%), bidis (0.9%), and dissolvable tobacco (0.8%).

During 2011–2012, among middle school students, for current electronic cigarette use, significant increases were observed overall (0.6% to 1.1%) and among females (0.4% to 0.8%), males (0.7% to 1.5%), and Hispanics (0.6% to 2.0%) (Table). For hookahs, a significant increase was observed among Hispanics (1.7% to 3.0%).

During 2011–2012, among high school students, for electronic cigarette use, significant increases were observed overall (1.5% to 2.8%) and among females (0.7% to 1.9%), males (2.3% to 3.7%), non-Hispanic whites (1.8% to 3.4%), and Hispanics (1.3% to 2.7%). For hookahs, significant increases were observed overall (4.1% to 5.4%) and among non-Hispanic whites (4.3% to 6.1%). For cigars, a significant increase in use was observed among non-Hispanic blacks (11.7% to 16.7%).

## Editorial Note

The findings in this report indicate that during 2011–2012 significant increases occurred in current use of nonconventional tobacco products, such as electronic cigarettes and hookahs, among middle and high school students; in addition, an increase in cigar use occurred among non-Hispanic black high school students. During this same period, overall current use of some tobacco products, such as bidis and kreteks, significantly decreased. These findings indicate that more efforts are needed to monitor and prevent the use of both conventional and nonconventional tobacco products among youths.

During 2011–2012, cigar use increased significantly among non-Hispanic black high school students to 16.7%, more than doubling the 2009 estimate (6). Further, cigar use among high school males (16.7%) was approximately double that of high school females (8.4%) and similar to cigarette use among high school males (16.3%). Cigars include traditional premium cigars as well as cigarillos and “little cigars,” which are similar to cigarettes in terms of appearance, but depending on their weight, can be taxed at lower rates and legally sold with certain flavors that are banned from cigarettes (7). Youths are known to have higher rates of cigar use than adults, which might be related to the lower price of some cigars (e.g., cigarillos and “little cigars”) relative to cigarettes, or the marketing of flavored cigars that might appeal to youths (8). Significant increases also were observed in overall use of current electronic cigarettes (9) and hookahs. Current use of electronic cigarettes doubled among middle and high school females, middle school males, and Hispanic high school students. Among non-Hispanic white high school students, this increase was slightly less than double (1.8% to 3.4%), and among high school males, this increase was slightly more than 60% (2.3 to 3.7). For current hookah use, an increase of more than 75% (1.7% to 3.0%) was observed for Hispanic middle school students; among high school students, an overall increase of more than 30% (4.1% to 5.4%) was observed, but for non-Hispanic whites, this increase was more than 40% (4.3% to 6.1%). The increase in use of electronic cigarettes and hookah tobacco could be attributed to low price, an increase in marketing, availability, and visibility of these products, and the perception that these tobacco products might be “safer” alternatives to cigarettes. Cigars, electronic cigarettes, hookah tobacco, and certain other new types of tobacco products are not currently subject to FDA regulation. FDA has stated it intends to issue a proposed rule that would deem products meeting the statutory definition of a “tobacco product” to be subject to the Federal Food, Drug, and Cosmetic Act.¶

What is already known on this topic?

Nearly 90% of adult smokers began smoking by age 18 years.

What is added by this report?

Although decreases in the use of certain tobacco products (bidis and kreteks) have been observed, current cigar use has increased among non-Hispanic black high school students (11.7% to 16.7%), and the use of nonconventional products, such as electronic cigarettes, have increased among middle school (0.6% to 1.1%) and high school (1.5% to 2.8%) students.

What are the implications for public health practice?

Current use of cigars and nonconventional tobacco products need to be monitored at local, state, and national levels. This is especially true for nonconventional tobacco products and specific population subgroups. To reduce tobacco use among youths, national and state tobacco control programs can continue to implement evidence-based strategies, including those that will work in coordination with the Food and Drug Administration to regulate the manufacture, distribution, and marketing of tobacco products.

The findings in this report are subject to at least six limitations. First, data were only collected from youths who attended either public or private schools and might not be generalizable to all middle and high school-aged youths. Second, data were self-reported; thus, the findings are subject to recall and response bias. Third, current tobacco use was defined by including students who responded to questions about at least one of the 10 tobacco products but might have had missing responses to any of the other tobacco products that were assessed; missing responses were considered as nonuse, which might have resulted in conservative estimates. Fourth, in 2012, the question wording for bidis and kreteks was modified, and cigar brand examples were added to the heading and ever cigar use question of the survey; therefore, any observed changes in prevalence estimates across years might be attributed in part to these wording modifications. Fifth, the NYTS overall response rate of 73.6% in 2012 and 72.7% in 2011 might have resulted in nonresponse bias, even after adjustment for nonresponse. Finally, estimates might differ from those derived from other youth surveillance systems, in part because of differences in survey methodology, survey type and topic, and age and setting of the target population. However, overall relative trends are similar across the various youth surveys (1).

Effective, population-based interventions for preventing tobacco use among youths are outlined in the Surgeon General’s report (1) and the World Health Organization’s MPOWER package (10). Interventions include increasing the price of all tobacco products, implementing 100% comprehensive smoke-free laws and policies in workplaces and public places, warning about the dangers of all tobacco use with tobacco use prevention media campaigns, increasing access to help quitting, and enforcing restrictions on all tobacco product advertising, promotion, and sponsorship. Interventions are best implemented as part of comprehensive tobacco control programs, which are effective in decreasing tobacco use in the United States (2). Full implementation of comprehensive tobacco control programs at CDC-recommended funding levels, in coordination with FDA regulations of tobacco products, would be expected to result in further reductions in tobacco use and changes in social norms regarding the acceptability of tobacco use among U.S. youths (1,2,10).

## Figures and Tables

**TABLE:** Percentage of middle and high school students currently using* tobacco products, by school level, sex, race/ethnicity, and product type — National Youth Tobacco Survey, United States, 2011 and 2012

					Sex							
		
	Total				Females				Males			
			
	2011		2012		2011		2012		2011		2012	
						
School level/Product type	%	(95% CI)	%	(95% CI)	%	(95% CI)	%	(95% CI)	%	(95% CI)	%	(95% CI)
**Middle school**												
Tobacco†	7.5	(6.5–8.8)	6.7	(5.8–7.7)	5.9	(4.7–7.4)	5.6	(4.7–6.7)	9.0	(7.9–10.3)	7.8	(6.7–9.0)
Cigarettes	4.3	(3.5–5.2)	3.5	(2.8–4.3)	4.0	(3.1–5.2)	3.2	(2.5–4.0)	4.5	(3.7–5.5)	3.8	(3.0–4.7)
Cigars	3.5	(2.8–4.2)	2.8	(2.4–3.4)	2.5	(1.9–3.4)	2.4	(1.9–3.2)	4.3	(3.4–5.4)	3.2	(2.7–3.8)
Smokeless tobacco	2.2	(1.8–2.7)	1.7	(1.3–2.1)	1.4	(1.0–2.0)	1.2	(0.8–1.6)	3.0	(2.3–3.8)	2.2	(1.7–2.9)
Pipes	2.2	(1.7–2.9)	1.8	(1.4–2.3)	1.8	(1.3–2.5)	1.7	(1.3–2.3)	2.7	(2.1–2.5)	1.9	(1.4–2.4)
Bidis	1.7	(1.3–2.2)	0.6	(0.5–0.7)§	1.4	(1.0–1.9)	0.4	(0.3–0.7)§	1.9	(1.4–2.6)	0.7	(0.5–1.0)§
Kreteks	1.1	(0.9–1.4)	0.5	(0.4–0.7)§	0.9	(0.6–1.3)	0.4	(0.3–0.7)§	1.3	(1.0–1.6)	0.6	(0.4–0.9)§
Hookahs	1.0	(0.8–1.4)	1.3	(1.0–1.7)	1.0	(0.6–1.6)	1.0	(0.7–1.4)	1.1	(0.7–1.5)	1.5	(1.1–2.2)
Snus	0.9	(0.6–1.2)	0.8	(0.6–1.0)	0.8	(0.5–1.2)	0.6	(0.4–0.9)	1.0	(0.6–1.4)	1.0	(0.7–1.4)
Dissolvable tobacco	0.3	(0.2–0.4)	0.5	(0.4–0.8)§	0.3	(0.2–0.5)	0.4	(0.2–0.6)	0.3	(0.1–0.5)	0.7	(0.4–1.1)¶
Electronic cigarettes	0.6	(0.4–0.9)	1.1	(0.9–1.5)§	0.4	(0.2–0.7)	0.8	(0.6–1.1)§	0.7	(0.4–1.3)	1.5	(1.1–2.1)§
**High school**												
Tobacco†	24.3	(22.1–26.6)	23.3	(21.6–25.2)	19.0	(17.0–21.1)	18.1	(16.2–20.1)	29.4	(26.6–32.4)	28.3	(26.2–30.6)
Cigarettes	15.8	(13.7–18.1)	14.0	(12.5–15.7)	13.8	(11.7–16.2)	11.7	(10.2–13.4)	17.7	(15.2–20.4)	16.3	(14.5–18.3)
Cigars	11.6	(10.5–12.7)	12.6	(11.4–13.9)	7.4	(6.3–8.6)	8.4	(7.2–9.8)	15.7	(14.3–17.2)	16.7	(15.0–18.5)
Smokeless tobacco	7.3	(5.9–9.0)	6.4	(5.5–7.5)	1.6	(1.2–2.2)	1.5	(1.1–2.1)	12.9	(10.4–15.9)	11.2	(9.5–13.0)
Pipes	4.0	(3.4–4.6)	4.5	(4.0–5.2)	2.8	(2.2–3.4)	3.2	(2.7–3.9)	5.1	(4.3–6.0)	5.8	(5.0–6.7)
Bidis	2.0	(1.6–2.5)	0.9	(0.7–1.1)§	1.0	(0.7–1.4)	0.5	(0.3–0.7)§	2.9	(2.3–3.7)	1.3	(1.0–1.7)§
Kreteks	1.7	(1.4–2.0)	1.0	(0.8–1.2)§	0.8	(0.6–1.2)	0.5	(0.3–0.7)§	2.4	(1.9–2.9)	1.5	(1.1–1.9)§
Hookahs	4.1	(3.4–5.0)	5.4	(4.6–6.3)§	3.5	(2.8–4.4)	4.5	(3.7–5.4)	4.8	(3.7–6.1)	6.2	(5.3–7.3)
Snus	2.9	(2.3–3.7)	2.5	(2.0–3.0)	0.8	(0.5–1.1)	0.9	(0.7–1.3)	5.1	(3.9–6.6)	3.9	(3.2–4.9)
Dissolvable tobacco	0.4	(0.3–0.6)	0.8	(0.6–1.0)§	0.1	(0.1–0.4)	0.6	(0.4–0.9)¶	0.6	(0.4–1.0)	1.0	(0.8–1.4)
Electronic cigarettes	1.5	(1.2–2.0)	2.8	(2.3–3.5)§	0.7	(0.5–1.0)	1.9	(1.5–2.4)§	2.3	(1.7–3.1)	3.7	(2.9–4.8)§

	Race/Ethnicity															
	
	White, non-Hispanic				Black, non-Hispanic				Hispanic				Other race, non-Hispanic			
				
	2011		2012		2011		2012		2011		2012		2011		2012	
								
School level/Product type	%	(95% CI)	%	(95% CI)	%	(95% CI)	%	(95% CI)	%	(95% CI)	%	(95% CI)	%	(95% CI)	%	(95% CI)
**Middle school**																
Tobacco†	6.2	(5.1–7.4)	5.1	(4.2–6.3)	8.5	(6.6–10.9)	7.7	(5.9–10.1)	11.5	(10.2–13.1)	10.5	(8.6–12.8)	6.1	(3.8–9.9)	3.1	(1.7–5.4)
Cigarettes	3.8	(2.8–5.1)	3.1	(2.4–4.0)	3.6	(2.6–5.0)	2.6	(1.7–4.0)	6.7	(5.6–8.0)	5.4	(4.2–7.1)	3.4	(2.0–5.8)	1.7	(0.8–3.6)¶
Cigars	2.3	(1.7–3.0)	1.6	(1.2–2.0)	5.7	(4.3–7.4)	5.0	(3.8–6.6)	6.1	(4.9–7.4)	4.9	(3.8–6.4)	1.6	(0.8–3.2)	1.5	(0.7–3.1)¶
Smokeless tobacco	2.3	(1.8–2.9)	1.6	(1.1–2.2)	1.0	(0.5–2.1)	0.6	(0.3–1.3)¶	2.9	(2.3–3.6)	2.4	(1.7–3.4)	2.4	(1.2–4.8)	1.4	(0.7–3.1)¶
Pipes	1.5	(1.1–2.2)	1.2	(0.8–1.7)	1.3	(0.8–2.1)	1.2	(0.6–2.2)¶	5.0	(4.2–6.1)	3.7	(2.7–5.1)	2.5	(1.2–5.0)	0.5	(0.2–1.1)¶
Bidis	1.0	(0.7–1.5)	0.3	(0.2–0.5)¶	1.9	(1.1–3.2)	0.6	(0.4–1.0)	3.5	(2.6–4.6)	1.2	(0.8–1.8)§	1.2	(0.5–2.8)	0.7	(0.2–2.4)¶
Kreteks	0.6	(0.4–0.6)	0.3	(0.2–0.5)	0.9	(0.5–1.6)	0.2	(0.1–0.7)¶	2.5	(2.0–3.3)	1.0	(0.6–1.7)§	1.8	(0.7–4.3)	0.7	(0.2–2.4)¶
Hookahs	0.9	(0.6–1.4)	0.8	(0.6–1.2)	0.9	(0.5–1.7)	0.9	(0.4–1.8)¶	1.7	(1.2–2.3)	3.0	(2.2–4.1)§	0.1	(0.0–0.5)	0.3	(0.1–1.6)¶
Snus	1.0	(0.7–1.4)	0.7	(0.5–1.0)	0.6	(0.2–1.3)	0.4	(0.1–0.9)¶	1.0	(0.6–1.5)	1.1	(0.7–1.7)	0.7	(0.2–2.5)	0.4	(0.1–2.8)¶
Dissolvable tobacco	0.2	(0.1–0.5)	0.4	(0.2–0.7)¶	0.4	(0.1–1.2)	0.5	(0.2–1.5)¶	0.2	(0.1–0.5)	1.0	(0.6–1.6)¶	0.4	(0.1–2.4)	0.1	(0.0–0.5)¶
Electronic cigarettes	0.6	(0.4–1.0)	0.9	(0.6–1.3)	0.4	(0.2–1.0)	1.1	(0.6–2.2)¶	0.6	(0.4–1.1)	2.0	(1.4–2.9)§	0.7	(0.2–2.6)	0.3	(0.1–0.8)¶
**High school**																
Tobacco†	26.6	(23.6–29.8)	24.6	(22.3–27.0)	18.9	(15.6–22.8)	22.6	(19.7–25.8)	23.8	(21.2–26.5)	22.5	(19.5–25.6)	13.9	(10.5–18.3)	13.7	(9.9–18.8)
Cigarettes	17.6	(14.7–20.9)	15.4	(13.2–17.8)	10.6	(7.6–14.6)	9.6	(7.6–12.0)	15.8	(13.9–17.8)	14.3	(12.0–16.9)	8.9	(6.2–12.5)	8.7	(5.9–12.5)
Cigars	12.1	(10.7–13.6)	12.2	(10.8–13.8)	11.7	(9.8–13.9)	16.7	(14.4–19.3)§	11.3	(9.8–13.1)	12.4	(10.6–14.4)	5.7	(4.0–8.1)	6.3	(4.4–9.0)
Smokeless tobacco	9.2	(7.4–11.5)	8.1	(6.9–9.5)	3.0	(1.8–5.1)	2.2	(1.5–3.2)	5.1	(3.8–6.8)	5.1	(3.8–6.8)	4.0	(2.4–6.6)	3.4	(2.3–5.2)
Pipes	3.5	(2.9–4.4)	4.5	(3.8–5.4)	2.4	(1.5–3.8)	2.9	(1.8–4.5)	6.3	(5.2–7.7)	6.2	(5.2–7.4)	3.4	(1.7–6.6)	2.4	(1.4–3.9)¶
Bidis	1.4	(1.0–2.0)	0.7	(0.5–1.0)§	2.0	(1.2–3.2)	0.8	(0.4–1.7)¶	3.7	(2.9–4.8)	1.4	(0.9–2.2)§	1.8	(1.0–3.4)	0.4	(0.2–1.1)¶
Kreteks	1.4	(1.0–2.0)	1.1	(0.8–1.5)	1.3	(0.8–2.2)	0.6	(0.3–1.1)¶	2.5	(1.9–3.3)	0.9	(0.6–1.4)§	2.0	(1.0–4.0)	0.3	(0.1–0.7)¶
Hookahs	4.3	(3.4–5.4)	6.1	(5.2–7.2)§	1.7	(0.9–3.0)	2.1	(1.6–2.9)	5.1	(4.1–6.3)	6.6	(5.1–8.5)	4.8	(2.5–9.0)	2.5	(1.5–4.1)¶
Snus	3.7	(2.8–4.9)	3.3	(2.6–4.2)	0.7	(0.3–1.5)	0.6	(0.3–1.1)¶	2.3	(1.7–3.1)	1.8	(1.3–2.5)	1.7	(0.7–3.8)	0.8	(0.4–1.6)¶
Dissolvable tobacco	0.3	(0.1–0.5)	0.7	(0.5–0.9)¶	0.3	(0.1–1.2)	0.8	(0.4–1.3)¶	0.8	(0.5–1.3)	1.4	(1.0–2.1)	0.6	(0.1–2.9)	0.5	(0.2–1.2)¶
Electronic cigarettes	1.8	(1.3–2.4)	3.4	(2.7–4.2)§	0.8	(0.3–1.7)	1.1	(0.7–1.9)¶	1.3	(0.8–2.1)	2.7	(1.9–3.8)§	0.6	(0.3–1.2)	2.2	(0.9–5.8)¶

**Abbreviation:** CI = confidence interval.

* Current use of cigarettes was determined by asking, "During the past 30 days, on how many days did you smoke cigarettes?" Current use of cigars was determined by asking, "During the past 30 days, on how many days did you smoke cigars, cigarillos, or little cigars?" Current use of smokeless tobacco was determined by asking, "During the past 30 days, on how many days did you use chewing tobacco, snuff, or dip?" Current use of a pipe was determined by asking, "During the past 30 days, on how many days did you smoke tobacco in a pipe?" In 2011, current use of bidis and kreteks was determined by asking, "During the past 30 days, on how many days did you smoke bidis?" and "During the past 30 days, on how many days did you smoke kreteks?" In 2012, current use of bidis and kreteks was determined by asking, "During the past 30 days, which of the following products (bidis and kreteks) have you used on at least 1 day?" Current use of hookahs, snus, dissolvable tobacco, and electronic cigarettes was determined by asking, "During the past 30 days, which of the following products (hookah, snus, dissolvable tobacco, and electronic cigarettes) have you used on at least 1 day?"

† Includes use for ≥1 day in the past 30 days of any of the following: cigarettes, cigars, smokeless tobacco, tobacco pipes, bidis, kreteks, hookahs, snus, dissolvable tobacco, or electronic cigarettes.

§ Difference between 2011 and 2012 was statistically significant by t-test (p<0.05).

¶ Data are statistically unreliable because sample size <50 or relative standard error >0.3 on at least 1 year's data; therefore, no t-test was performed.

## Footnotes

* The question to assess past 30 day use of bidis changed between 2011 and 2012. In 2011, the bidis question was “In the past 30 days, on how many days did you smoke bidis?” Students selected among “0 days,” “1 or 2 days,” “3 to 9 days,” “10 to 19 days,” “20 to 29 days,” or “all 30 days.” In 2012, the bidis question was “In the past 30 days, which of the following products have you used on at least one day?” Students could select different products, of which “bidis (small brown cigarettes wrapped in a leaf)” was a possible selection. This change might have affected the results for bidis.

† The question to assess past 30 day use of kreteks changed between 2011 and 2012. In 2011, the kreteks question was “In the past 30 days, on how many days did you smoke kreteks?” Students selected among “0 days,” “1 or 2 days,” “3 to 9 days,” “10 to 19 days,” “20 to 29 days,” or “all 30 days.” In 2012, the bidis question was “In the past 30 days, which of the following products have you used on at least one day?” Students could select different products, of which “clove cigarettes (kreteks)” was a possible selection. This change might have affected the results for kreteks.

§ The heading for the cigar section of the questionnaire changed between 2011 and 2012. In 2011, the heading was “Cigars.” In 2012, the heading was “Cigars, cigarillos, or little cigars, such as Black and Milds, Swisher Sweets, Dutch Masters, White Owl, or Phillies Blunts,” and the question on ever use of cigars also included brand names. This change might have affected the results for cigars.

¶ FDA has expressed its intent to assert jurisdiction over all tobacco products. Additional information available at http://www.reginfo.gov/public/do/eAgendaViewRule?pubId=201304&RIN=0910-AG38.
