# Erratum

**Published:** 2013-11-22

## Vol. 62, No. 45

In the report, “Tobacco Product Use Among Middle and High School Students — United States, 2011 and 2012,” an error occurred in the first paragraph on pages 893 and 894. The sixth sentence of that paragraph should read, “During the same period, significant decreases occurred in bidi^*^ and kretek^†^ use among middle and high school students.”

